# Spectroscopy detects skeletal muscle microvascular dysfunction during onset of sepsis in a rat fecal peritonitis model

**DOI:** 10.1038/s41598-022-10208-w

**Published:** 2022-04-15

**Authors:** Paulina M. Kowalewska, Justin E. Kowalewski, Stephanie L. Milkovich, Richard J. Sové, Lin Wang, Shawn N. Whitehead, Christopher G. Ellis

**Affiliations:** 1 Department of Medical Biophysics, University of Western Ontario, London, ON Canada; 2 Department of Psychiatry, University of Western Ontario, London, ON Canada; 3 Department of Biomedical Engineering, Johns Hopkins School of Medicine, Baltimore, MD USA; 4 Department of Anatomy and Cell Biology, University of Western Ontario, London, ON Canada

**Keywords:** Biophysics, Physiology, Biomarkers, Diseases, Pathogenesis

## Abstract

Sepsis is a dysregulated host inflammatory response to infection potentially leading to life-threatening organ dysfunction. The objectives of this study were to determine whether early microvascular dysfunction (MVD) in skeletal muscle can be detected as dynamic changes in microvascular hemoglobin (MVHb) levels using spectroscopy and whether MVD precedes organ histopathology in septic peritonitis. Skeletal muscle of male Sprague–Dawley rats was prepared for intravital microscopy. After intraperitoneal injection of fecal slurry or saline, microscopy and spectroscopy recordings were taken for 6 h. Capillary red blood cell (RBC) dynamics and SO_2_ were quantified from digitized microscopy frames and MVHb levels were derived from spectroscopy data. Capillary RBC dynamics were significantly decreased by 4 h after peritoneal infection and preceded macrohemodynamic changes. At the same time, low-frequency oscillations in MVHb levels exhibited a significant increase in Power in parts of the muscle and resembled oscillations in RBC dynamics and SO_2_. After completion of microscopy, tissues were collected. Histopathological alterations were not observed in livers, kidneys, brains, or muscles 6 h after induction of peritonitis. The findings of this study show that, in our rat model of sepsis, MVD occurs before detectable organ histopathology and includes ~ 30-s oscillations in MVHb. Our work highlights MVHb oscillations as one of the indicators of MVD onset and provides a foundation for the use of non-invasive spectroscopy to continuously monitor MVD in septic patients.

## Introduction

Sepsis is a complicated and highly heterogeneous condition with respect to etiology and progression. Early diagnosis and treatment initiation affect septic patient outcomes^1,2^ and if not achieved, patients develop organ dysfunction^3–7^. However, diagnosis in the early stages of sepsis is difficult; clinical signs are non-specific, covering variations in temperature, elevated heart rate, hypotension, decreased capillary refill and variation in leukocyte counts^8^. As well, biomarkers of sepsis, including C-reactive protein, procalcitonin and hyperlactatemia, are present in a range of inflammatory conditions^8^ and more complex biomarker panels would have to be developed for precise diagnosis^9^. Likewise, scoring systems used to evaluate morbidity and predict mortality at bedside are not specific to sepsis and interpretations are complicated in patients with pre-existing conditions^10^. Thus, development of additional tools to detect the onset and progression of sepsis is needed.

One of the earliest pathophysiological characteristics of experimental sepsis is microvascular dysfunction (MVD), which occurs prior to systemic hypotension^11^. MVD in skeletal muscle was first observed by intravital video microscopy (IVVM) in a rat peritonitis model where local microvascular derangements were evidenced by changes in functional capillary density marked by stopped red blood cell (RBC) flow^12^. It was proposed that impairment of the microvascular autoregulatory system causes uncoupling of local O_2_ delivery from O_2_ demand in sepsis, thereby increasing the risk of tissue hypoxia, tissue damage and progression to organ dysfunction^13–15^. If this hypothesis is correct, evidence of MVD would precede markers of tissue injury and clinical signs of sepsis.

Whether septic MVD precedes tissue histopathology in various organs is debated. In a mouse model of sepsis induced by cecal ligation and puncture (CLP), histologic analysis showed tissue injury is apparent by 6 h after infection^16,17^. Specifically, inflammatory and structural changes were noted in the lungs, livers and kidneys. However, these studies did not focus on MVD progression. Thus, one of our goals was to build upon this research by determining whether skeletal muscle MVD precedes histopathological alterations in vital organs in a rat model of feces-induced peritonitis (FIP). Fecal peritonitis is an established model of sepsis with rat mortality of 33% by 22 h post-induction, despite antibiotic treatment and resuscitation^18^.

While there are no clinically established methods to monitor MVD, several approaches have been used in small trials on septic patients, most prominently sidestream darkfield (SDF) video microscopy of the sublingual microvasculature^19^ and near-infrared spectroscopy (NIRS) of skeletal muscle^20^. These methods were key to showing that deteriorated tissue perfusion is associated with survival in sepsis^20–23^. Despite technical limitations that do not permit continuous monitoring and yield recordings of short duration with motion induced image blurring, as well as difficulty in resampling the same area of tissue for imaging, SDF has shown significantly altered microvascular perfusion and flow dynamics in septic patients^19^. NIRS also proved useful in detecting decreased baseline tissue oxygenation and its recovery after ischemic challenge in sepsis^20^, although others reported no difference in baseline tissue O_2_ saturation between septic patients and healthy volunteers^24,25^. A method that overcomes limitations in data resampling and permits continuous microvascular flow monitoring by generating real-time data would be ideal to establish the onset of MVD and evaluate responses to treatment in sepsis.

To detect MVD in our rat model, we applied dual wavelength (*λ*) IVVM to make precise measurements of capillary RBC dynamics and O_2_ transport in the extensor digitorum longus (EDL) with repeated measurements from the same tissue regions over 6 h after induction of peritonitis. Capillary RBC O_2_ saturation (SO_2_) was measured from the optical density (OD) ratio of O_2_-dependent to O_2_-independent (isosbestic) *λ* of light transmitted through individual RBCs in EDL capillaries^11^. However, IVVM is an invasive technique and, as with SDF, requires considerable time for analysis. Thus, we were interested in applying a method that has potential to be non-invasive and detects changes in skeletal muscle perfusion that reflect MVD in sepsis in real-time. To achieve this, we took advantage of the spectral properties of hemoglobin and used continuous wave spectroscopy (CWS) to measure changes in OD by capturing light transmitted through the skeletal muscle for the same fields of view recorded with IVVM. The EDL is a relatively thin muscle, allowing for transillumination with negligible light scatter. This permitted the use of multiple isosbestic *λ* throughout the visible spectrum in contrast to the use of more deeply penetrating near-infrared *λ* in human patients. Changes in OD measured at isosbestic *λ* are proportional to changes in microvascular hemoglobin (MVHb) levels in the volume of tissue being sampled^26^. We hypothesized that spectroscopically measured MVHb dynamics in healthy muscle reflect regulation of blood flow to maintain adequate tissue oxygenation. Accordingly, we expect that MVD development at the onset of sepsis will be detected as altered MVHb dynamics. We further set out to determine if this MVD precedes organ pathology. Indeed, we show that microvascular failure can be detected spectroscopically in skeletal muscle remote to the infectious locus and is apparent before histopathologic alterations in various organs, including brain inflammation, in our rat peritonitis model.

## Methods

### Animals

All animal procedures were approved by the University of Western Ontario Animal Care Committee of the University Council on Animal Care (Protocol #2015–105) and all methods were carried out in accordance with the guidelines and regulations set by the committee. Twenty male Sprague–Dawley rats were obtained from Charles River (Charles River Laboratories, Wilmington, MA). The rats were communally housed (2–3 rats per cage) under constant temperature and humidity with a 12-h light/dark cycle and allowed ad libitum access to food and water. At the time of use, the animals were approximately 7 weeks old. The ARRIVE guidelines were followed for reporting in vivo research^27^.

### Surgical preparation and physiological monitoring

Animals were randomized to a FIP or saline injection group. The rats were anesthetized with an intraperitoneal (IP) injection of sodium pentobarbital (65.64 mg/kg; Ceva Santé Animale, Libourne, Nouvelle-Aquitaine, France). The body temperature was maintained at 36.5–37.5 °C with a heat lamp and monitored using a rectal thermometer (Acorn Temp JKT; Cole-Parmer Instrument Co., Montreal, QC, Canada).

The left common carotid artery was cannulated with polyethylene 50 tubing (I.D. 0.58 mm, O.D. 0.965 mm; Becton Dickinson and Company, Sparks, MD, USA) that was pressurized with 0.9% NaCl (normal saline; pH 5.5, Na 154 mmol/L, Cl 154 mmol/L, Osmolarity 308 mOsmol/L; Baxter corporation, Mississauga, ON, Canada) with 1% heparin (Sandoz, Holzkirchen, Upper Bavaria, Germany). This allowed for monitoring of blood pressure and heart rate through a pressure transducer (Edwards Lifesciences) and Blood Pressure Analyzer (Digi-Med BPA-400; Micro-Med Inc., Louisville, KY, USA). The trachea was cannulated to allow mechanical ventilation (Inspira Advanced Safety Ventilator; Harvard Apparatus, Holliston, MA, USA) with a flow-controlled O_2_/N_2_ mixture using standard settings for rats that were minimally modified based on arterial blood gas values (CG4 + cartridge, Abbott Point of Care, IL, USA; VetScan iSTAT 1 Handheld Analyzer, Abaxis, Union City, CA, USA). The right external jugular vein was cannulated using silastic tubing (I.D. 0.64 mm, O.D. 1.19 mm; Carpinteria, CA, USA). A continuous infusion of normal saline through the venous line was used for minimal fluid resuscitation (0.5 mL/h) and maintenance anesthesia. Depth of anesthesia was assessed by the palpebral reflex.

The right EDL was isolated, and its tendon was secured with a silk suture and severed distal to the ligature as previously described^11,28^. Animals randomized to the FIP group were injected with approximately 0.5 mL of a fecal slurry (approximately 0.25 g of autologous feces in normal saline) intraperitoneally through a small midline incision. The control animals received 0.5 mL of normal saline. The animal was placed on the microscope stage in the right lateral position. Suture on the EDL tendon was secured to the stage, keeping the muscle at its in situ length and orientation. The EDL was bathed with Plasma-Lyte A (37 ºC, pH 7.4, Na 140 mmol/L, Cl 98 mmol/L, K 5 mmol/L, Mg 1.5 mmol/L, Acetate 27 mmol/L, Gluconate 23 mmol/L, Osmolarity 294 mOsmol/L; Baxter corporation, Mississauga, ON, Canada), isolated with an O_2_-impermeable polyvinylidene chloride film (SaranWrap; Dow Chemical Company, Midland, MI, USA), and gently compressed with a glass coverslip. The EDL was given 30 min to stabilize on the microscope stage and its microcirculation was recorded over a period of 6 h (Supplementary Fig. S1 online). Gases and lactate levels in arterial blood taken from the carotid artery were measured at baseline and between each imaging period (VetScan iSTAT 1 Handheld Analyzer). Microvascular perfusion and responses are stable in our surgically exposed EDL preparation^29^.

### Dual wavelength intravital video microscopy

**Figure 1:**
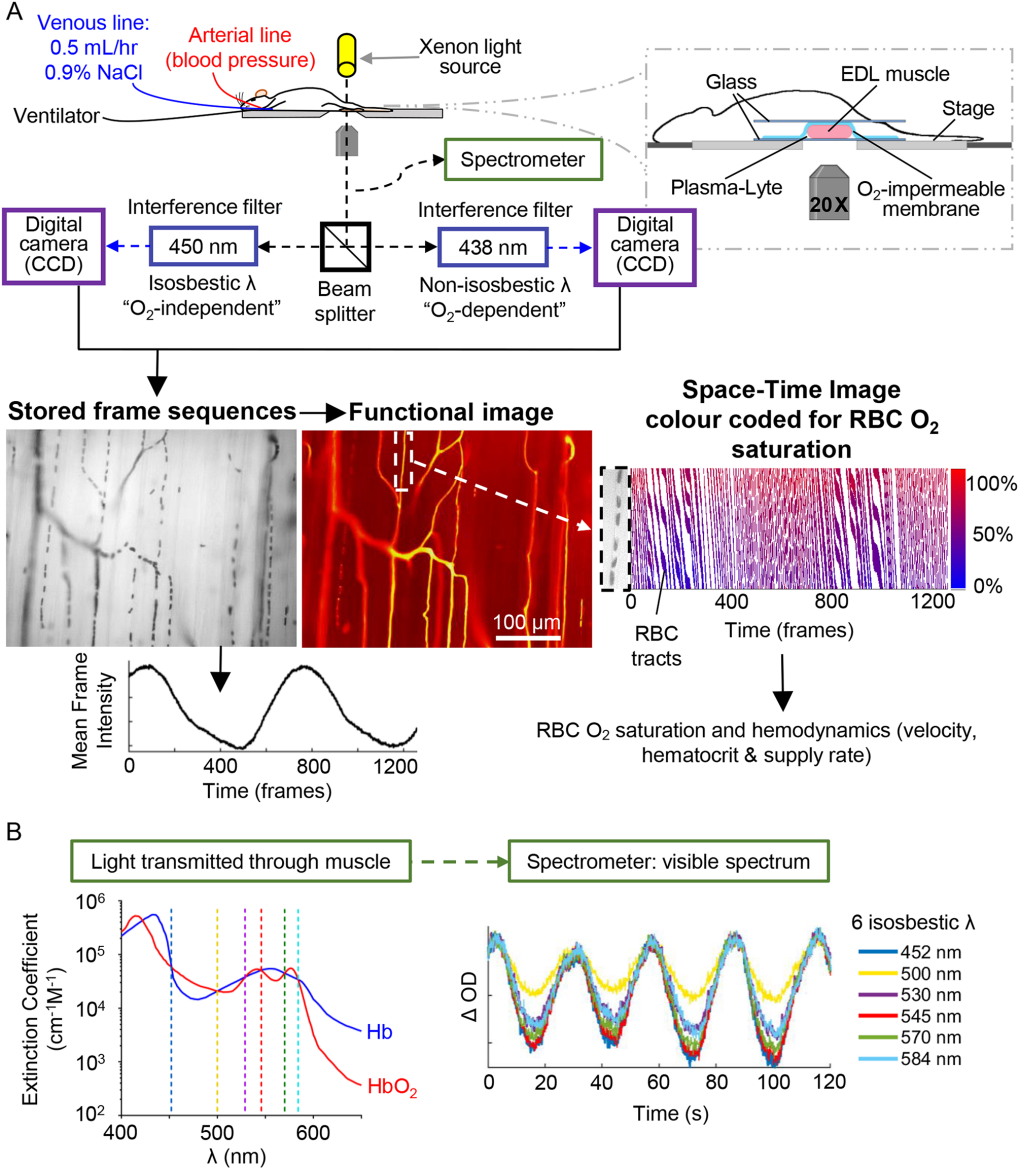
Stage setup and image analysis. Intravital microscopy (**A**): Rats were positioned on a stage with the isolated extensor digitorum longus (EDL) muscle in the light path. Light transmitted through the muscle was split between two cameras and captured at two *λ* that allow for calculation of RBC O_2_ saturation based on spectral properties of fully oxygenated and deoxygenated hemoglobin. Functional images showing the passage of RBCs through capillaries were derived from the captured frame sequences, and space–time images were generated for capillary segments showing RBC transit as tracts colour coded for O_2_ saturation. RBC dynamics were calculated from these space–time images. As well, the mean intensity of each captured frame at 450 nm was measured. Spectroscopy (**B**): the intensities of 6 *λ* isosbestic for fully oxygenated/deoxygenated hemoglobin were measured from light transmitted through the muscle for each field of view after microscopy recordings.

The EDL was transilluminated with a 75-W xenon light source and imaged on an Olympus IX-81 inverted microscope (Olympus Corporation, Tokyo, Kanto, Japan). Light was split between two 12-bit monochrome charge-coupled device cameras (QImaging Rolera-XR FAST 1394 digital cameras; Surrey, BC, Canada) with a 50/50 beam splitter (MAG Biosystems DC2 Dual-Channel, Full-Field, Simultaneous-Imaging System; Exton, PA, USA). The beam splitter was fitted with interference filters for 438-nm *λ* (hemoglobin O_2_-dependent) and 450-nm *λ* (isosbestic; hemoglobin O_2_-independent), allowing for simultaneous dual *λ* frame capture (696 × 520-pixel resolution; 12-bit depth; 21 frames/sec) by the two cameras with a 20 X objective. The IVVM images were recorded for 1 min (~ 8 fields of view/rat) and stored as raw PNG files (one per frame) using custom acquisition software (Neovision, Prague, Czech Republic) and processed using in-house software written in MATLAB (MathWorks, Natick, MA, USA). The microscopy setup and image analysis are outlined in Fig. 1.

### Continuous wave spectroscopy

Each individual 1-min IVVM recording was followed by a spectrometer recording of the same field of view. A USB2000 Fiber Optic Spectrometer (Ocean Optics Inc, Dunedin, FL, USA) was attached through a fiber optic C-Mount-MIC eyepiece adaptor for SMA 905 Fibers (Ocean Optics Inc, Dunedin, FL, USA) mounted on the microscope. Using the OOIBase32 software (Ocean Optics Inc, Dunedin, FL, USA), transilluminated light (*λ* ranging from 450 to 650 nm) from the exposed EDL was collected for 2 min with the spectrometer (10–11 recordings/sec). This generated approximately 1200 individual light intensity profiles for each *λ* within this range.

### Tissue collection and processing

After completion of intravital imaging in another cohort of rats, arterial blood was collected and mixed with 3% acetic acid and 1% crystal violet in a 5∶44∶1 ratio. Hemocytometer counts were averaged from 6 separate samples. Rats were overdosed with pentobarbital and perfused transaortically with phosphate-buffered saline, followed by 4% paraformaldehyde (PFA). Transaortic perfusion-fixation allowed for collection of the brain, left extensor digitorum longus, left lateral lobe of the liver and kidneys but not lungs or hearts which were bypassed by the solutions.

The muscles, livers and kidneys were immersion-fixed in 4% PFA for 3 days, followed by dehydration and embedding in paraffin. The thin-sectioned tissues (5 μm) were stained with hematoxylin and eosin (H&E) and assessed for tissue damage using an upright microscope (Olympus BX-50, Hitachi SXGA ECCO HV-F22 camera, Northern Eclipse software, Olympus Corp.).

### Brain immunohistochemistry

Brains were post-fixed in 4% PFA for 24 h at 4 ºC and transferred into 30% sucrose solution at 4 ºC. Brains were frozen, sectioned at 30 µm and every 6^th^ coronal slice (spanning the whole brain) was immunostained for rat MHC class II with anti-rat RT1B (clone OX-6; BD Biosciences, Mississauga, ON, Canada) to detect microglial activation^30^. Specifically, free-floating sections were treated with 1% H_2_O_2_ and blocked with horse serum (Vector Laboratories, Inc., Burlingame, CA, USA). The sections were incubated with anti-rat RT1B in blocking serum overnight at 4 °C, followed by 1-h incubation with biotinylated 2° antibody (BA-2000; Vector Laboratories, Inc.) in serum at room temperature. Sections were then incubated with avidin–biotin complex (Vector Laboratories, Inc.) for 1 h followed by 0.05% diaminobenzidine. After washing, sections were mounted on slides with 0.3% gelatin and air-dried, followed by dehydration, clearing in xylenes, and mounting with DePex mounting medium (Electron Microscopy Science, Hatfield, PA, USA). After examination under high magnification, stitched images of brain sections were acquired using an upright microscope (Nikon Eclipse Ni-E, Nikon DS Fi2 color camera, NIS Elements Imaging, Mississauga, ON, Canada).

### Offline analysis

Functional capillary density (capillaries/mm) was determined by counting capillaries that crossed three horizontal lines on a 450 μm by 340 μm field of view and capillary RBC flow was categorized as continuous, intermittent (stopped for > 3 s at least once or reversed flow) or completely stopped during a 30-s segment of the recording. Capillary location and geometry were determined from functional images generated from the video sequence^31,32^. Analysis of RBC SO_2_ and flow through capillaries in each field of view was performed as previously described^33^. Briefly, software extracted frame-by-frame light intensity data from the centre line of in-focus capillaries over the length of the sample to generate a space–time image (STI) of the passage of RBCs through each capillary. To calculate the OD of each RBC, the incident light intensity was determined from the light intensity of the plasma gaps between RBCs in a capillary. OD of each RBC at both *λ* was calculated as the ratio of the intensity of transmitted light through the RBC to the incident intensity. RBC hemoglobin SO_2_ was determined by calculating the 438 nm/450 nm OD ratio with constants derived from in vivo calibration and displayed as SO_2_ colour-coded STIs. Capillary hemodynamics were calculated from the analysis of the 450 nm STI yielding frame-by-frame data for capillary RBC velocity (μm/s), lineal density (RBC/mm)/hematocrit (%) and RBC supply rate (RBC/s)^29^. Of note, capillaries with stopped flow were assigned a velocity and supply rate of 0.

The variation in hemoglobin content in the volume of tissue (1 mm thickness of muscle encompassing multiple capillary modules as well as arteriolar and venular trees) associated with each field of view was determined from the acquired video sequence and from the CWS spectroscopy data. The mean intensity of all pixels in each video frame of the 1-min sequence was calculated for the 450-nm *λ*. The resulting mean frame intensity (MFI) time series was processed to generate the change in OD relative to the first time point, i.e. ΔOD_450_(t). The CWS spectroscopy data was processed in the same manner for all *λ* values yielding an array of ΔOD_*λ*_(t). For this study, ΔOD values for the 6 isosbestic *λ* (452 nm, 500 nm, 530 nm, 545 nm, 570 nm and 584 nm; bandpass 2 nm) were calculated; note, these are the isosbestic points in the visible spectrum (Fig. 1B). At these isosbestic *λ*, the extinction coefficients are independent of the hemoglobin SO_2_ and hence only reflect dynamic changes in MVHb content in the sample volume. We averaged signals from the 6 isosbestic points to increase the accuracy of our MVHb measurements. Fourier analysis was applied to the MFI and spectroscopy data to quantify the Power of low-frequency oscillations observed in MVHb content. Specifically, the Fourier transforms of the MFI and spectroscopy data over time were approximated using the built-in fast Fourier transform algorithm in MATLAB (MathWorks, Natick, MA, USA). The Power was quantified as the square of the signal amplitude and the peak Power was calculated by taking the maximum of the Power spectrum in the 0.03–0.06 Hz range (corresponding periods of 16 s – 32 s). The peak Power of these low-frequency oscillations from MFI and CWS recordings was compared between FIP and control groups.

### Statistics

Analysis was performed using GraphPad Prism version 7.00, (GraphPad Software; La Jolla, CA, USA). A two-way ANOVA with a Bonferroni post-hoc test was conducted to evaluate differences between the control and FIP groups over the three imaging periods. All values are reported as mean (standard error of the mean; SEM). Statistical significance was defined as *P* < 0.05.

## Results

### Animal characteristics and physiological parameters during intravital microscopy

The weights of control and FIP animals used in the experiment did not significantly differ (0.174 kg (0.007) vs 0.182 kg (0.003), *P* = 0.322). After surgical preparation for IVVM and during the 6-h imaging period, none of the animals died. Macrohemodynamics and physiological parameters were monitored during the study to determine the timing of their deterioration (Table 1). MAP remained stable in the control group through the 6-h imaging period. MAP was stable in the FIP group from 0.5–4 h after infection but was significantly decreased during the 4.5–6 h imaging period compared with control animals (*P* < 0.0001; Table 1). Thus, macrovascular changes occurred closer to the experimental endpoint in the FIP group. Lactate levels, a marker of tissue hypoperfusion, were significantly increased in rats with FIP compared with controls at 4.5 h (*P* = 0.0015; Table 1) and 6 h after infection (*P* < 0.0001). At endpoint, the feces-injected animals had systemic leukocyte counts that were significantly lower than the saline-injected animals (2.8 (1.1) vs 6.6 (0.9) × 10^6^ cells/mL, respectively, *P* < 0.0001). Based on their decreased MAP, increased lactate levels (> 2 mmol/L)^34^, and leukopenia^35,36^, rats with FIP in our study were progressing towards a septic state at the experimental endpoint. We were, however, able to maintain stable PaO_2_ values in both groups through the imaging period, which did not significantly differ between the control and FIP groups (Table 1). Also, the arterial partial pressure of O_2_ to fraction of inspired O_2_ ratio (PaO_2_/FiO_2_) did not significantly change over time (Table 1), indicating that ventilator settings used in this study did not cause acute respiratory distress syndrome, which can affect RBC SO_2_.

**Table 1:** Mean arterial pressure, lactate, PaO_2_ and PaO_2_/FiO_2_ ratios. Data are reported as mean (SEM) and assessed using two-way ANOVA with Bonferroni correction. *P < 0.05 compared with control, *n* = 10 rats.

Group	Time point (hrs after infection)	Mean arterial pressure (mmHg)	Lactate (mmol/L)	PaO_2_ (mmHg)	PaO_2_/FiO_2_
Saline-injected control	0.5–2 h	85.3 (2.7)	1.00 (0.12)	106.0 (4.6)	335 (12)
	2.5–4 h	81.4 (1.8)	0.84 (0.11)	97.0 (4.9)	324 (23)
	4.5–6 h	78.2 (1.9)	0.84 (0.09)	97.6 (2.1)	317 (6)
	Endpoint	N/A	0.98 (0.13)	99.9 (6.8)	341 (31)
Feces-induced peritonitis	0.5–2 h	86.8 (2.6)	1.41 (0.28)	105.4 (6.2)	340 (16)
	2.5–4 h	79.3 (2.7)	1.04 (0.15)	94.3 (4.4)	321 (19)
	4.5–6 h	60.8 (3.1)*	1.85 (0.24)*	91.4 (7.1)	293 (30)
	Endpoint	N/A	2.75 (0.27)*	96.9 (5.7)	329 (27)

### Functional capillary density in skeletal muscle declines by 4 h after induction of fecal peritonitis

**Figure 2:**
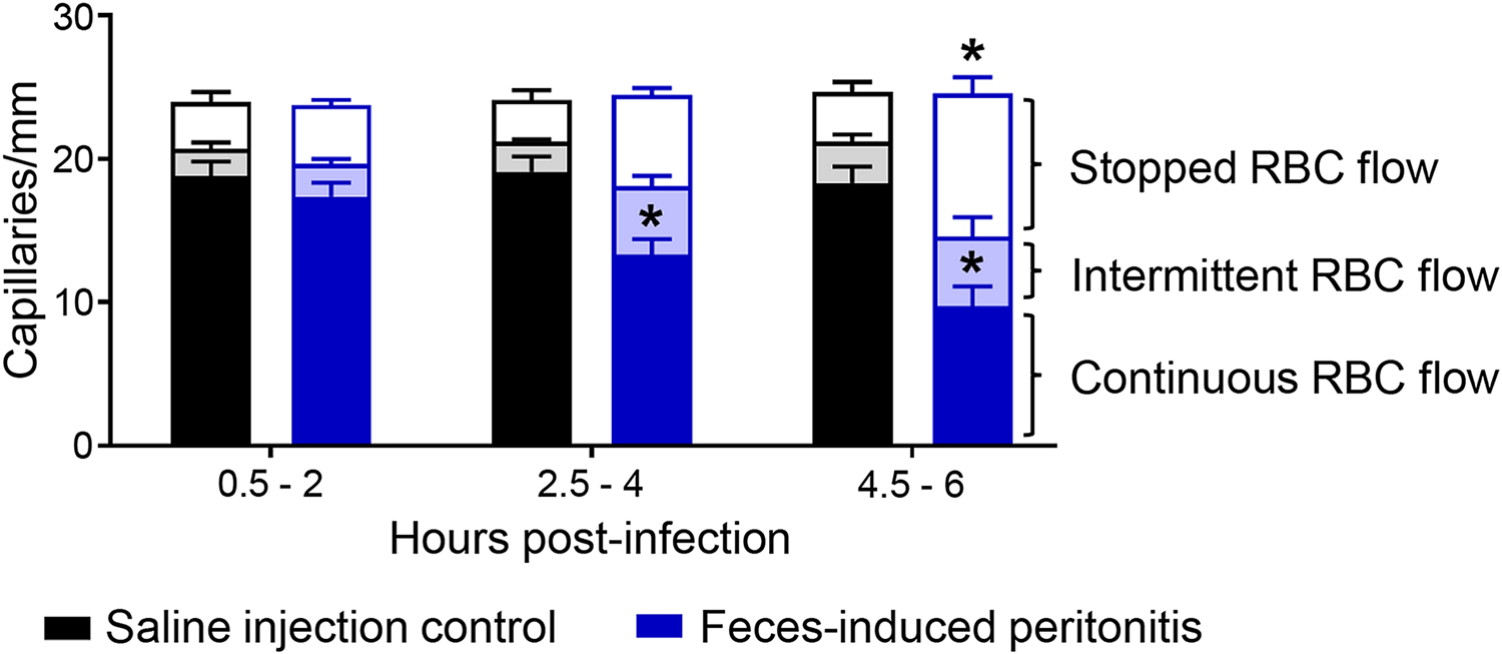
Functional capillary density deteriorates in extensor digitorum longus after induction of fecal peritonitis. Red blood cell (RBC) flow through capillaries was categorized as continuous flow, intermittent flow, or stopped flow over a 30-s period in each video recording at 0.5–2 h, 2.5–4 h and 4.5–6 h after induction of peritonitis with a fecal slurry or injection of saline (control). Values are expressed as mean (SEM) and assessed using two-way ANOVA with Bonferroni correction, *n* = 10 rats with approximately 8 fields of view averaged per rat for each time point, *P < 0.05 compared with control.

**Figure 3:**
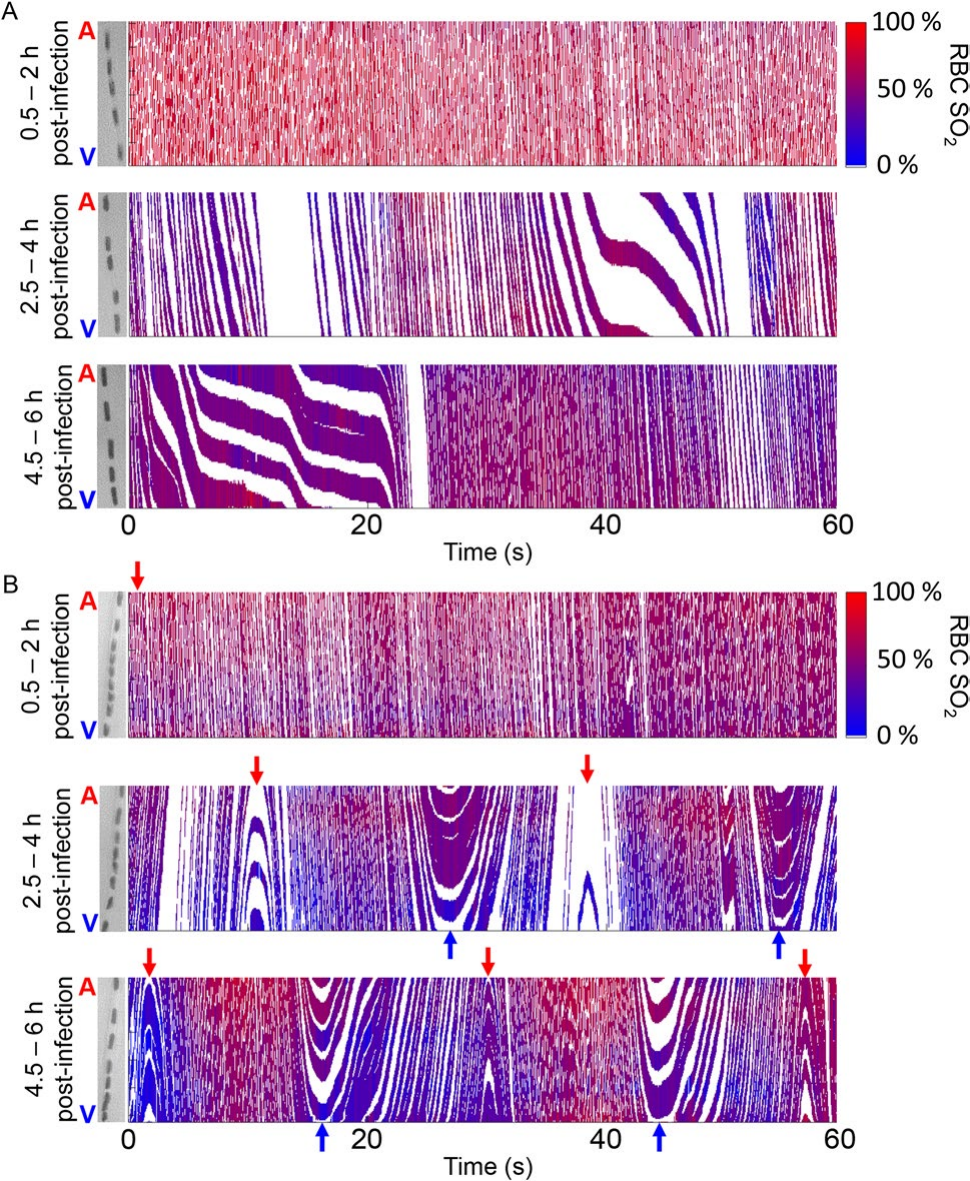
Intermittent capillary red blood cell (RBC) flow. Space–time images display RBC flow in a capillary segment recorded at three timepoints over the 6-h imaging period in a rat with feces-induced peritonitis. Tracts represent passage of RBCs and are colour coded for O_2_ saturation. Intermittent flow emerges 2.5–4 h after infection and appears as cyclical stopped-flow (**A**) or reverse flow (**B**). Red arrows indicate flow from the arteriolar end of the capillary; blue arrows indicate flow from the venular end. Note the high-velocity flow of RBCs with low O_2_ saturations from the venous end 4.5–6 h after induction of fecal peritonitis (**B**).

The number of capillaries with continuously flowing RBCs was significantly decreased in the FIP group compared with control animals during the 2.5–4 h imaging period (*P* = 0.0002; Fig. 2) as capillaries progressed to intermittent (Fig. 3) and stopped-flow states. The decline in continuously flowing capillaries in animals with FIP continued 4.5–6 h after infection (*P* < 0.0001) while the number of capillaries that had stopped flow significantly increased (*P* < 0.0001). This reduced capillary perfusion was in concert with increased lactate levels (Table 1) and preceded significant decrease in MAP, highlighting a mismatch between microhemodynamic and macrohemodynamic deterioration in the fecal peritonitis model of sepsis.

### Capillary red blood cell dynamics in skeletal muscle deteriorate early in feces-induced peritonitis

**Figure 4:**
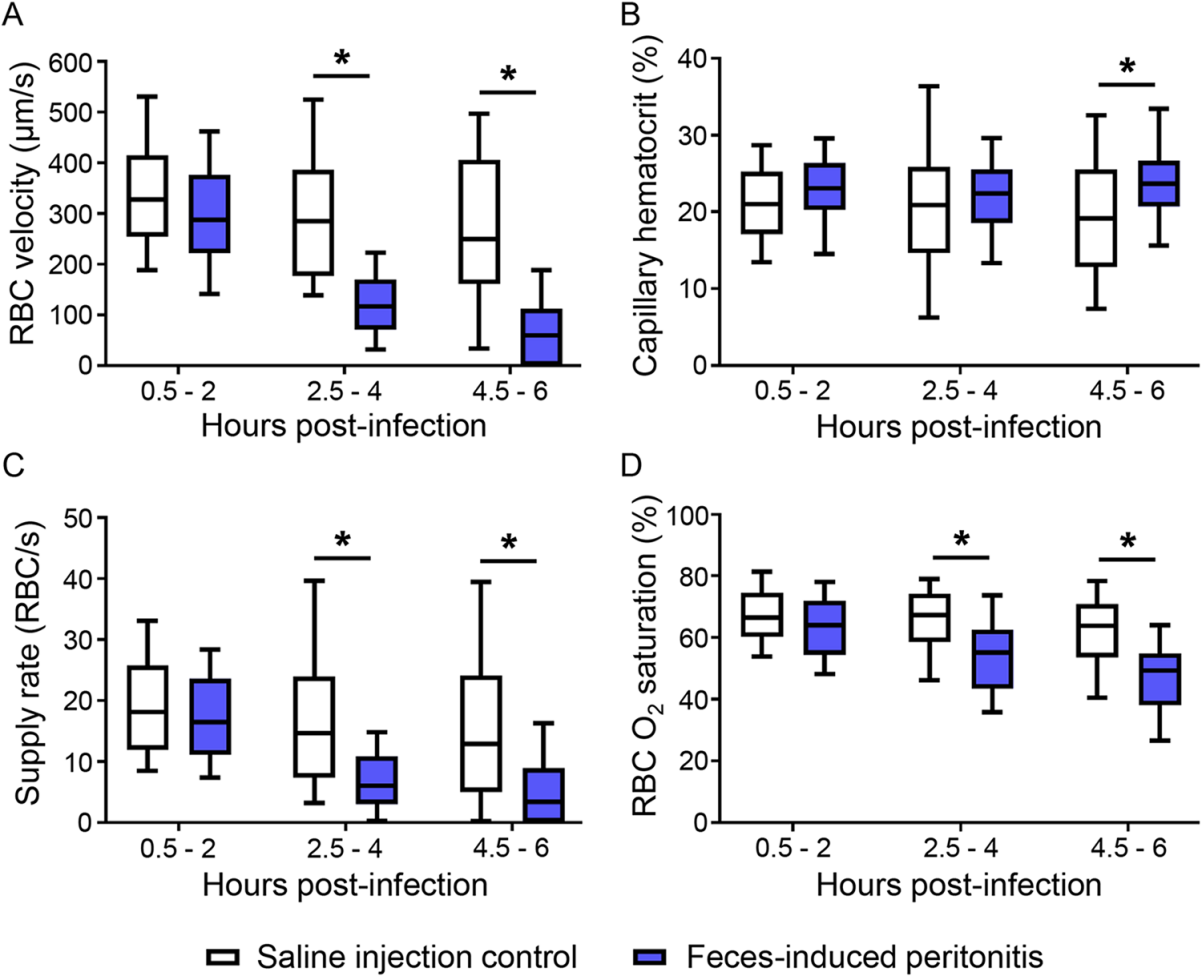
Capillary red blood cell (RBC) dynamics and O_2_ saturation in skeletal muscle deteriorate early in feces-induced peritonitis model of sepsis. RBC velocity (**A**), hematocrit (**B**), supply rate (**C**) and O_2_ saturation (**D**) were derived from intravital video microscopy of the extensor digitorum longus recorded 0.5–2 h, 2.5–4 h and 4.5–6 h after induction of peritonitis. Data were pooled from approximately 8 fields of view from each rat in the saline control and feces-induced peritonitis groups, and presented as box and whiskers (median, 10th to 90th percentile), *n* = 10 rats, two-way ANOVA with Bonferroni correction, *P < 0.05 compared with control.

Altered capillary RBC dynamics were evident as early as 2.5–4 h after induction of fecal peritonitis. At baseline, RBC velocity was not significantly different between control rats and animals with FIP (Fig. 4A). However, capillary RBC velocities were significantly decreased in the FIP group 2.5–4 h (*P* < 0.0001) and 4.5–6 h (*P* < 0.0001) after infection. The distribution of capillary RBC velocities was similar in control and FIP animals in the first imaging period (Fig. 4A) but shifted towards lower velocities in the FIP group by 4.5–6 h, while the velocities in the control group became more widely distributed—an effect of surgical exposure of the muscle. Of note, we did not see increased numbers of hyperemic capillaries by 6 h after fecal injection as was observed in rats 24 h post-CLP^11^, indicating that MVD manifests differently as sepsis progresses. Capillary hematocrit was similar between the two groups during baseline recordings and 2.5–4 h after infection (Fig. 4B). At 4.5–6 h after infection, hematocrit in the capillaries that were still flowing in the FIP animals was slightly higher compared to controls (*P* = 0.0033). However, the hematocrit values collected during this 4.5–6 h time point did not change from baseline hematocrit in the FIP group (repeated two-way ANOVA with Tuckeys multiple comparisons test, *P* = 0.5808). Thus, overall, capillary hematocrit in the FIP group appeared stable. While RBC supply rates were similar between the two groups at baseline (Fig. 4C), there was a significant decline with FIP 2.5–4 h (*P* < 0.0001) and 4.5–6 h (*P* < 0.0001) after infection. RBC SO_2_ was also similar at baseline between the two groups (Fig. 4D) and significantly decreased in FIP animals in the second (*P* < 0.0001) and third (*P* < 0.0001) imaging period.

**Figure 5:**
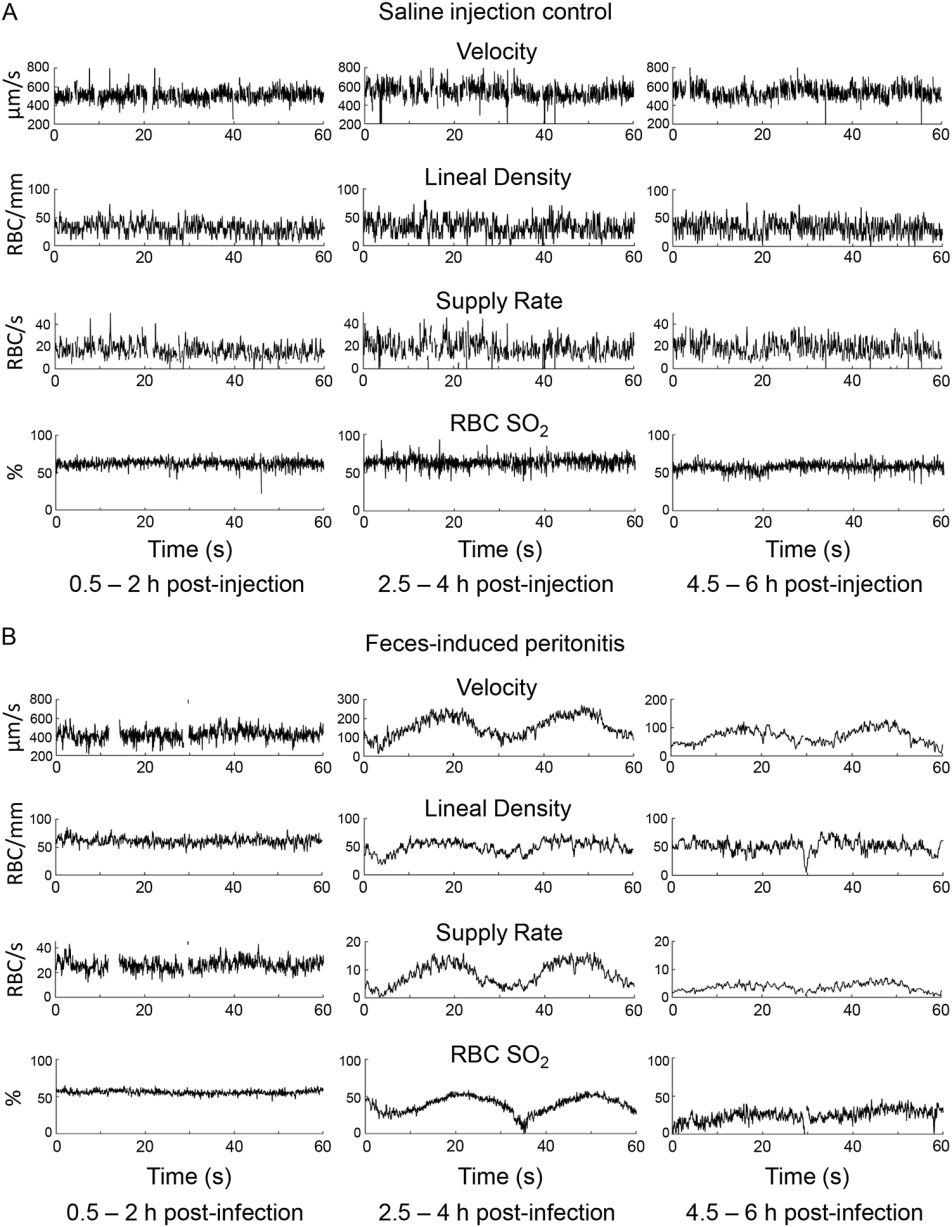
Low-frequency oscillations in capillary red blood cell (RBC) flow dynamics and O_2_ saturation emerge in rat skeletal muscle after induction of fecal peritonitis. Microhemodynamic (RBC velocity, lineal density (reflective of hematocrit) and supply rate) and RBC O_2_ saturation (SO_2_) measurements from intravital video microscopy recordings are shown for a capillary from a control rat (**A**) and a feces-injected rat (**B**). The imaging was performed for 6 h after intraperitoneal injection of normal saline (control) or fecal slurry (feces-induced peritonitis). RBC SO_2_ measurements are based on the optical density ratio of the O_2_-dependent to O_2_-independent *λ*.

By 2.5–4 h after fecal injection, oscillatory flow patterns begun to emerge in capillary RBC velocity, hematocrit and supply rate at roughly 2 cycles/min (Fig. 5B). These oscillations were not readily observed in control animals, which had overall steady levels of RBCs flowing with some variability on a second-to-second basis (Fig. 5A). The ~ 30-s cycles also appeared in RBC SO_2_ in capillaries of animals with fecal peritonitis (Fig. 5B), suggesting the microvasculature lost the ability to maintain constant SO_2_. In areas of the muscle characterized by oscillatory RBC flow, some capillaries had completely stationary RBCs, indicating that dysfunction within a capillary module may be heterogeneous.

### Oscillations in microvascular hemoglobin levels, derived from spectroscopy data, indicate early microvascular dysfunction in skeletal muscle of rats with fecal peritonitis

**Figure 6:**
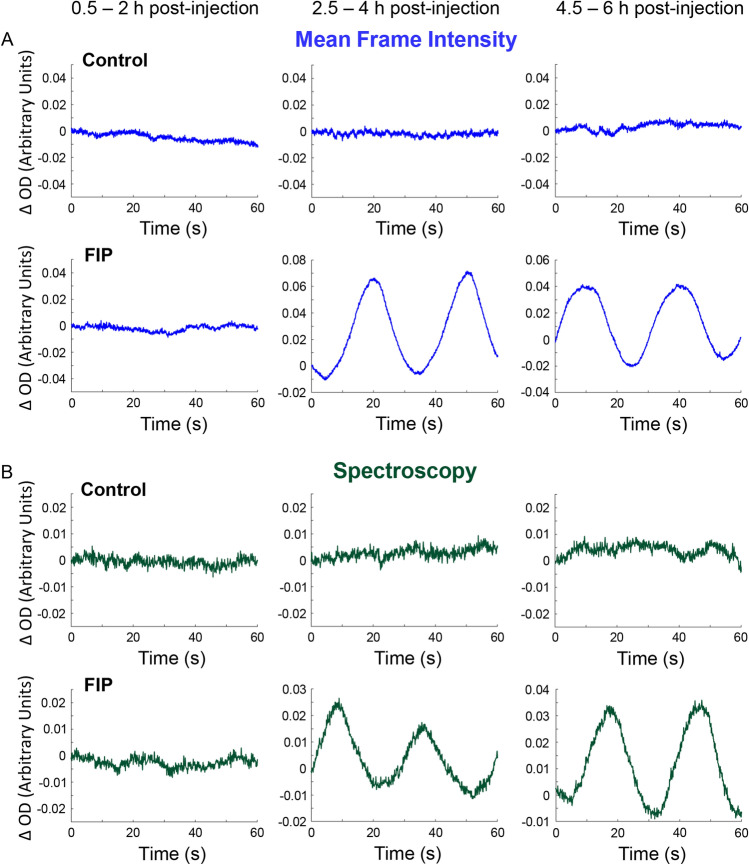
Optical spectroscopy detects low-frequency oscillations in microvascular hemoglobin levels in rat skeletal muscle after induction of fecal peritonitis. The change in optical density (ΔOD) at 450 nm *λ* was calculated from the mean frame intensities of intravital video microscopy recordings (**A**). The mean ΔOD of 6 isosbestic *λ* was derived from continuous wave spectroscopy measurements (**B**). Measurements were taken at three timepoints over 0.5–6 h after intraperitoneal injection of normal saline (control) or fecal slurry (FIP, feces-induced peritonitis). The ΔOD traces in **A** and **B** reflect microvascular hemoglobin content and are matched for an illustrative field of view from each group.

The EDL is a relatively thin muscle, allowing for transillumination with negligible light scatter. ODs were calculated from light intensity data of the captured IVVM frames and spectroscopy profiles of the EDL. OD at isosbestic *λ* = extinction coefficient × path length × [hemoglobin]. Since the extinction coefficient and path length through the muscle are constant, ΔOD reflects Δ[hemoglobin]. Through the 6-h imaging period, the mean ΔOD of 6 isosbestic *λ* captured with CWS from the muscle remained relatively stable at high frequencies in control animals, similar to the MFI from IVVM recordings. Graphs from a representative field of view are shown in top rows of Fig. 6A and B. In the animals with fecal peritonitis, however, vasomotion was detected as low-frequency oscillations by 2.5–4 h after infection in both MFI and CWS recordings (bottom rows in Fig. 6A,B).

**Figure 7:**
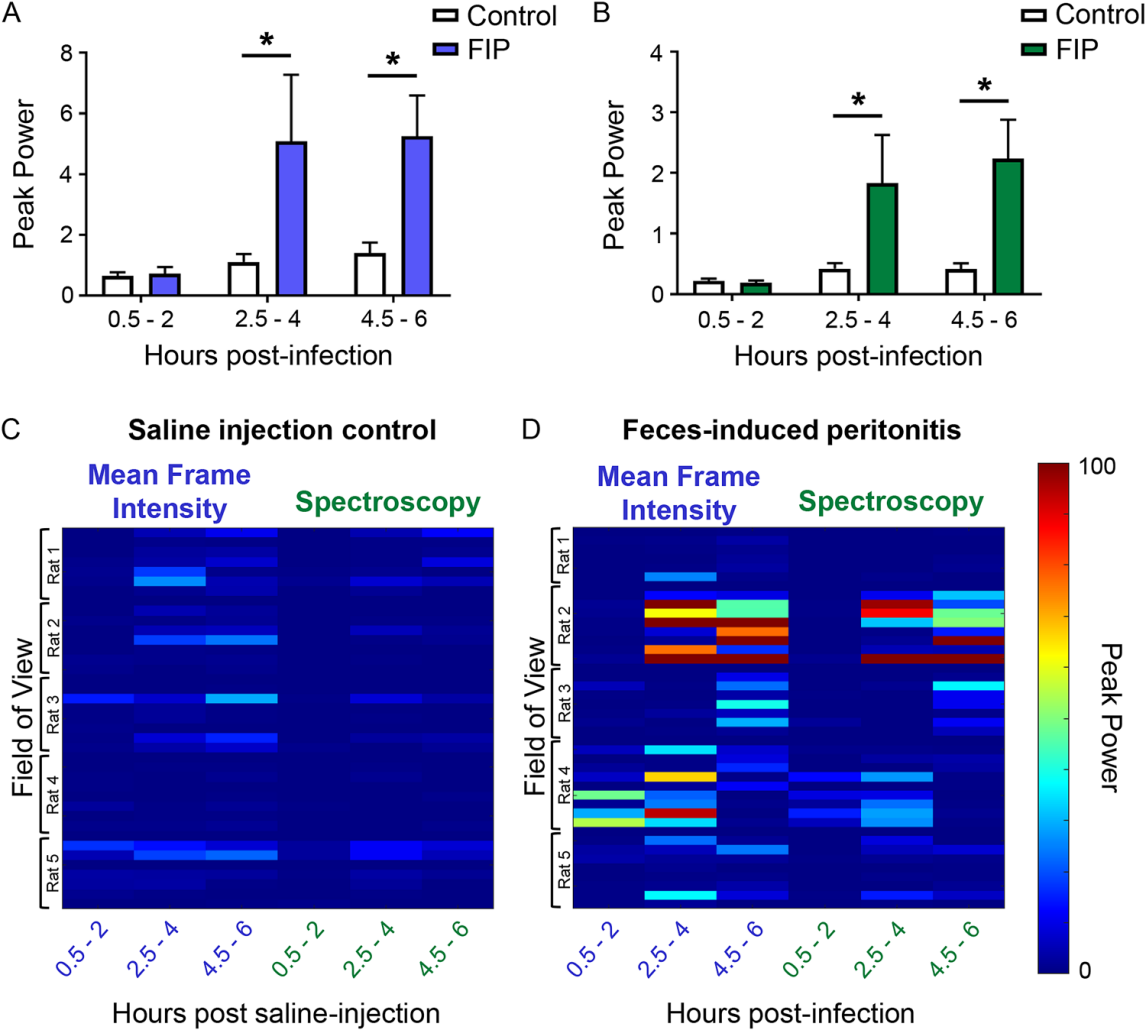
The Power of low-frequency oscillations in microvascular hemoglobin content is increased in skeletal muscle of rats with feces-induced peritonitis. Summary data show change in Power (amplitude^2^) of low-frequency oscillations in hemoglobin derived from mean frame intensity of intravital microscopy videos (**A**) and continuous wave spectroscopy measurements (**B**) recorded over 6 h after injection of normal saline or feces-induced peritonitis (FIP). Data are presented as mean (SEM) and assessed using two-way ANOVA with Bonferroni correction, *n* = 5 rats with approximately 8 fields of view/rat, *P < 0.05 compared with control. Heat maps for control (**C**) and FIP group (**D**) show the Power of low-frequency oscillations for each field of view resampled over the three imaging periods.

To summarize the type of data exemplified in Fig. 6 and indirectly Fig. 5, Fourier analysis was performed on the light intensity data collected with microscopy (MFI) and spectroscopy (CWS). Fourier analysis of the OD measurements from the MFI and CWS data exhibited a significant increase in Power at low frequencies in hemoglobin levels associated with vasomotion by 4 h after fecal injection (Fig. 7). Outlier analysis was performed using the ROUT method and 33 outliers out of 219 values were removed from MFI data over the three timepoints and 38 outliers out of 219 values were removed from the spectroscopy data. Fields of view that contained an outlier during the first, second or third imaging period were not used to preserve data matching across time. Power (amplitude^2^) of the low-frequency oscillations from in vivo MFI signals was significantly increased in the FIP group compared to the control group in the second (*P* = 0.0252) and third imaging period (*P* = 0.0312; Fig. 7A). Likewise, the Power of low-frequency oscillations from CWS data was significantly increased in the FIP group compared to controls in the second (*P* = 0.0498) and third imaging period (*P* = 0.0066; Fig. 7B). Of note, the average peak period of these oscillations in the FIP groups was 28.8 (2.7) seconds. The Power of these oscillations was displayed as a heat map across all the fields of view for control (Fig. 7C) and FIP (Fig. 7D) rats. The oscillations appeared in several fields of view from muscles of the FIP group during the 2.5–4.5 h imaging period and were particularly prominent in rat 2 and rat 4, as labelled in the figure (Fig. 7D). However, some fields of view from FIP rats did not exhibit these low-frequency oscillations (Fig. 7C,D), which may have resulted from substantially reduced flow in these tissue volumes. These low-frequency oscillations in the spectroscopy data closely reflected oscillations in RBC velocities, hematocrit, supply rates and SO_2_ in individual muscle capillaries of rats with fecal peritonitis (Fig. 5). Thus, oscillations in hemoglobin levels from the spectroscopy data appear to be one of the early indicators of microvascular derangement in sepsis.

### Histopathologic alterations are not apparent in organs from rats with feces-induced peritonitis at 6 h post-infection

**Figure 8:**
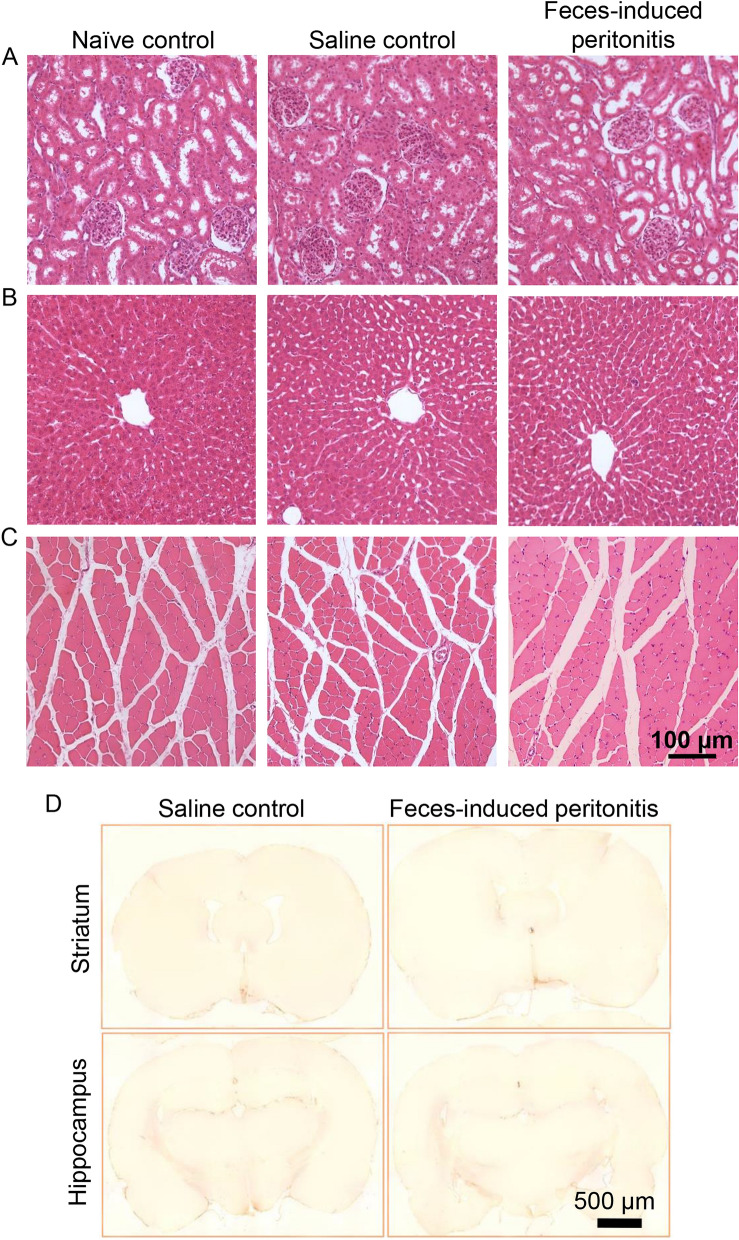
Histopathologic alterations and microglial activation were not observed in organs from rats at 6 h after induction of peritonitis. After completion of in vivo microscopy, rats underwent transaortic perfusion-fixation. Hematoxylin & eosin-stained cross-sections of the left kidney (**A**), liver (**B**) and left extensor digitorum longus (**C**) are shown. Naïve control animals were not subjected to intravital imaging. Coronal brain sections after staining with anti-MHC II antibody did not show microglial activation (**D**). Representative images from select areas of the brain are shown.

Organs were collected following perfusion-fixation after 6 h of IVVM and stained with H&E (Fig. 8A-C). The EDL muscles from animals with FIP did not exhibit increased tissue inflammatory cell infiltration. Erythrocyte trapping within the glomerular microvasculature did not rise above control levels. As well, livers from FIP and saline control animals did not exhibit excessive leukocyte accumulation and parenchymal cell architecture was not altered. No vacuolization of the hepatic parenchymal cells was noted in the two groups. These findings suggest that, in our rat FIP model of sepsis, microvascular dysfunction occurs despite lack of detectable organ histopathology in the early stages of the infection. Immunohistochemical labelling of brain sections for rat MHC class II did not show signs of pro-inflammatory microglial activation in brains of FIP rats with careful inspection under high magnification. While coronal slices spanning the entire brain were examined, sections encompassing the striatum and hippocampus are shown (Fig. 8D). Endothelin-1-induced brain infarct in a rat model served as positive control for this technique^30^.

## Discussion

Septic microvascular dysfunction manifests as decreased arteriolar responsivity^37^, increased stopped-flow capillaries, reduced capillary RBC supply rates, reduced RBC SO_2_, and increased capillary response time to hypoxia^13^, all tied to impaired microvascular autoregulation. We believe that high-amplitude, low-frequency oscillations in microvascular hemoglobin levels may also be one of the early indicators of microvascular failure in sepsis and reflect the onset of impaired microvascular autoregulation in the early stages of severe infection. Furthermore, we demonstrate that these oscillations can be detected non-invasively in skeletal muscle remote to the infectious locus. As well, we show that these oscillations precede macrohemodynamic deterioration and histopathologic alterations in vital organs. This work highlights the potential use of non-invasive spectroscopy techniques to continuously monitor MVD in the early stages of sepsis using easily accessible regions of the body such as skeletal muscle.

Several hypotheses have been put forth to explain the pathophysiological processes that lead to organ injury and dysfunction in sepsis. Our work centers on the idea that microvascular derangement happens early in sepsis, affecting O_2_ delivery and consequently instigating tissue injury. Maldistribution of RBC flow through capillary networks in septic rat skeletal muscle, manifested as increased flow heterogeneity with stopped- and fast-flow capillaries, leads to increased diffusion distance for O_2_ in the tissue^11^. Thus, the consequence of septic microvascular dysfunction is a mismatch between O_2_ demand and supply. Conversely, it is also believed that early mitochondrial dysfunction in sepsis diminishes O_2_ utilization and drives progression to multi-organ failure^38^. However, increased O_2_ extraction in septic rat skeletal muscle suggests that mitochondrial dysfunction does not occur in the early stage of sepsis in our animal model^11^. This is in line with our results, which show reduced RBC SO_2_ in skeletal muscle capillaries despite normal systemic PaO_2_ in the FIP rats.

Another proposed mechanism of tissue hypoperfusion in sepsis is blockage via accumulation of adherent leukocytes in capillaries and post-capillary venules, yet leukocyte-endothelial interactions in the EDL of septic rats were observed to be reduced 6–48 h after CLP^39^. Thus, microvascular obstruction by leukocytes is an unlikely cause of the reduced muscle perfusion we observed in our feces-injected rats. Microthrombi may also contribute to capillary plugging and disseminated intravascular coagulation is a common feature of sepsis^40^. In fact, stopped-flow capillaries in the EDL from septic mice 6–7 h after induction of fecal peritonitis are marked by P-selectin-dependent platelet adhesion and fibrin formation^41^. However, in our study, RBC supply rate and SO_2_ were significantly decreased by 4 h after fecal injection while the number of stopped-flow capillaries did not significantly increase till closer to the experimental endpoint. Thus, it appears that regulatory failure precedes any possible plugging of capillaries.

Tissue hypoperfusion in sepsis is believed to be tied to impaired signaling from capillaries to their supplying arterioles. It is clear that electrical communication between coupled vascular cells is diminished in early sepsis, evidenced by blunted conducted responses in mouse skeletal muscle^37,42–45^. Diminished communication between capillaries and arterioles in O_2_ demand–supply coupling may be based on ATP signalling as regulatory failure in sepsis was found to involve loss of O_2_ sensing in hypoxic capillaries with an impaired RBC O_2_-dependent efflux of ATP^13^. ATP release as RBCs desaturate^46^ is proposed to be a purinergic stimulus^47^ for conducted hyperpolarization and the resulting functional hyperemia^15^. ATP efflux is also induced by RBC deformation^48,49^ and, indeed, septic RBCs were found to be less deformable^50^. Taken together, these studies suggest that sepsis impairs conducted hyperpolarization along the capillary endothelium to the upstream arteriole, and along the arteriolar tree. We believe such impaired vascular autoregulation may underlie the observed loss of fine second-to-second control of hemoglobin delivery and the dominance of a fundamental low frequency of arteriolar vasomotion in our current study.

The goals of our study were to determine whether MVD can be detected with spectroscopy and whether spectroscopy signals qualitatively and temporally correspond to microhemodynamic changes and mean frame intensity oscillations observed with IVVM. Both methods detected temporal variability in MVHb levels in the early stages of fecal peritonitis, manifested as low-frequency OD oscillations by 4 h post-infection. The peak Power of these oscillations in spectroscopic measurements was significantly increased in FIP rats. However, in some areas of the muscle, no oscillations were observed, which may be due to capillary networks with RBC flow too low to generate a detectable oscillatory MVHb signal within the volume of tissue. This indicates that MVD is not entirely uniform across the muscle. Since the oscillations in capillary RBC flow synchronously spanned more than one 450 μm by 340 μm field of view and MVHb oscillations from spectroscopy data were derived from a volume of tissue spanning the entire EDL thickness, we believe rhythmic diameter changes occurred in larger arterioles.

The ~ 30-s microhemodynamic oscillations in the FIP group include periodically stopped flow, slower flow and reversed flow of RBCs within the vascular network. Alternating forward and reversed RBC flow through capillaries is due to pressure gradient changes in the microvascular network. In skeletal muscle, capillaries running between an arteriole and a venule are referred to as a capillary unit or module^51,52^. Of the capillaries supplied by a given terminal arteriole, roughly half drain into one venule and the other half (running in the opposite direction along muscle fibres) drain into another venule. Likewise, a given post capillary venule drains capillary modules from two or more terminal arterioles. This arrangement repeats along muscle fibres, such that a given fibre is supplied by multiple capillary modules^51^. These capillary modules are connected in series forming long columnar capillary structures, i.e. capillary fascicles^53^. The structure of the capillary fascicle appears to correspond to the muscle fascicle. Given the length of the capillary fascicle, many of the terminal arterioles supplying these modules originate from different branches of the arteriolar tree. Thus, for the microvasculature to maintain proper RBC flow direction in each capillary unit, blood pressures must be tightly controlled by coordinated regulation within multiple branches of the arteriolar tree. When regulation within the arteriolar tree is not coordinated, high pressure in one terminal arteriole may cause flow reversals in capillary modules normally supplied by adjacent terminal arterioles or low arteriolar pressures may result in flow reversals supplied by the venular tree. We observe such uncoordinated regulation as reversed flow of desaturated RBCs in muscle capillaries after induction of peritonitis (Fig. 3). This pathologic flow pattern contributed to the lower RBC SO_2_ observed in our FIP rats. Since this lack of coordination of arteriolar constriction or dilation would affect modular flow over long distances, it possibly contributed to the observed ~ 30-s oscillations in our IVVM and spectroscopy data—this will be an area for future study.

It has been actively debated whether MVD precedes tissue injury in sepsis, and our work aligns with the notion that it does. As the development of acute respiratory distress syndrome^54^, hepatic dysfunction^3^, and/or acute kidney injury^4,5^ is common in septic patients and skeletal muscle wasting in sepsis survivors can result in long-term mobility problems^6^, preclinical sepsis studies largely incorporate biochemical and histopathologic analysis. At 24 h after induction of fecal peritonitis in mice, considerable variability was observed in tissue injury between animals and even different organs in a given animal^55^. In some reports, tissue injury was detected as early as 6 h after cecal ligation and puncture in a mouse model^16,17^. Our model differs from these studies in that there is no necrotic intestinal tissue induced by puncture as an additional inflammatory source. Furthermore, our rats were not conscious through the 6-h study period, were supported on a ventilator and continuously provided intravenous fluids over the duration of IVVM. These factors may have contributed to the lack of observed histopathology in several organs of our feces-injected rats in the early stage of the infection. Notably, the severity of the infectious insult in different animal models is hard to calibrate and directly compare, and thus the strength of our study is that MVD analysis and histology were done in the same animals.

Septic patients may develop acute brain dysfunction, referred to as sepsis-associated encephalopathy, which is linked to greater mortality^56^, and sepsis survivors are at risk of long-term cognitive impairment^57^. The mechanisms that drive septic brain injury are not completely understood, and inflammation^58,59^, oxidative stress^60^, MVD^61^, and dynamic variability in MAP and arterial CO_2_ levels^62^ are suspected. Animal studies attempted to dissect the key inflammatory players in septic brains, with microglial activation as an important driver^63^, which has also been suggested in septic patients^59^. However, conclusions on the timing of microglial activation after induction of sepsis in animal models vary widely^64–71^. In our study, we examined inflammation in brains collected 6 h after induction of fecal peritonitis and found no evidence of microglial activation at this early time-point. It is important to understand whether peripheral MVD (such as in skeletal muscle) occurs before brain inflammation and if MVD monitoring could guide early intervention measures to reduce the risk of neurological dysfunction in sepsis survivors. Furthermore, it is unknown if MVD in the cerebrum itself causes brain damage, leading to acute loss of cognitive function or chronic long-term cognitive dysfunction.

Analogous to our spectroscopy approach to detect MVD preclinically, NIRS has been used to assess microvascular function in septic patients^25,72^. Our study supports the use of NIRS as a diagnostic tool for non-invasive continuous monitoring of MVD in sepsis. Furthermore, the analysis and interpretation of the oscillatory dynamics of MVHb signal in our study via direct comparison to in vivo microscopy observations could be applied to NIRS to garner more information from microvascular monitoring in critically ill patients. In fact, we have already begun monitoring skeletal muscle microvascular hemoglobin levels in the critically ill population using NIRS and have shown the feasibility of this approach with the presence of low frequency oscillations in some of the septic patients^26^.

Using a fecal peritonitis model of sepsis, the current study showed that dynamic changes in microvascular hemoglobin levels detected with spectroscopy correspond to microcirculatory deterioration observed with in vivo microscopy, and that MVD precedes macrohemodynamic changes and organ histopathology. This work provides a foundation for using non-invasive spectroscopy with deeper tissue penetrance, such as NIRS, as a diagnostic tool to detect and monitor MVD in septic patients. Spectroscopic detection of MVD may guide early sepsis screening and diagnostic pathways, allowing for prompt therapeutic interventions. By targeting microcirculatory preservation as well as restoration early, sepsis mortality and risk of long-term morbidity may be reduced, warranting further study in clinical populations.

## Supplementary Information

Supplementary Information.

## Data Availability

The datasets used and/or analysed in this study are available from the corresponding author on reasonable request.

## References

[1] Ferrer R (2014). Empiric antibiotic treatment reduces mortality in severe sepsis and septic shock from the first hour. Crit. Care Med..

[2] Leisman DE (2017). Survival benefit and cost savings from compliance with a simplified 3-hour sepsis bundle in a series of prospective, multisite, observational cohorts. Crit. Care Med..

[3] Brun-Buisson C, Meshaka P, Pinton P, Vallet B (2004). EPISEPSIS: a reappraisal of the epidemiology and outcome of severe sepsis in French intensive care units. Intensive Care Med..

[4] Poukkanen M (2013). Acute kidney injury in patients with severe sepsis in Finnish Intensive Care Units. Acta Anaesthesiol. Scand..

[5] Vincent J-L (2006). Sepsis in European intensive care units: results of the SOAP study. Crit. Care Med..

[6] Yende S (2016). Long-term quality of life among survivors of severe sepsis. Crit. Care Med..

[7] Polito A (2013). Pattern of brain injury in the acute setting of human septic shock. Crit. Care.

[8] Pirozzi N (2016). Sepsis: epidemiology, pathophysiology, classification, biomarkers and management. Emerg. Med. Trauma Surg. Care.

[9] Vincent J-L (2016). The clinical challenge of sepsis identification and monitoring. PLOS Med..

[10] van der Woude SW, van Doormaal FF, Hutten BA, Nellen JF, Holleman F (2018). Classifying sepsis patients in the emergency department using SIRS, qSOFA or MEWS. Neth. J. Med..

[11] Ellis CG, Bateman RM, Sharpe MD, Sibbald WJ, Gill R (2002). Effect of a maldistribution of microvascular blood flow on capillary O_2_ extraction in sepsis. Am. J. Physiol. Circ. Physiol..

[12] Lam C, Tyml K, Martin C, Sibbald W (1994). Microvascular perfusion is impaired in a rat model of normotensive sepsis. J. Clin. Invest..

[13] Bateman RM, Sharpe MD, Jagger JE, Ellis CG (2015). Sepsis impairs microvascular autoregulation and delays capillary response within hypoxic capillaries. Crit. Care.

[14] Ellis CG, Milkovich S, Goldman D (2012). What is the efficiency of ATP signaling from erythrocytes to regulate distribution of O_2_ supply within the microvasculature?. Microcirculation.

[15] Ellsworth ML (2009). Erythrocytes: oxygen sensors and modulators of vascular tone. Physiology.

[16] Dwivedi DJ (2016). Differential expression of PCSK9 modulates infection, inflammation, and coagulation in a murine model of sepsis. Shock.

[17] Mai SHC (2015). Delayed but not early treatment with DNase reduces organ damage and improves outcome in a murine model of sepsis. Shock.

[18] Rudiger A (2018). Heart rate elevations during early sepsis predict death in fluid-resuscitated rats with fecal peritonitis. Intensive Care Med. Exp..

[19] De Backer D (2013). Microcirculatory alterations in patients with severe sepsis: impact of time of assessment and relationship with outcome. Crit. Care Med..

[20] Creteur J (2007). The prognostic value of muscle StO_2_ in septic patients. Intensive Care Med..

[21] De Backer D, Creteur J, Preiser J-C, Dubois M-J, Vincent J-L (2002). Microvascular blood flow is altered in patients with sepsis. Am. J. Respir. Crit. Care Med..

[22] Sakr Y, Dubois M-J, De Backer D, Creteur J, Vincent J-L (2004). Persistent microcirculatory alterations are associated with organ failure and death in patients with septic shock. Crit. Care Med..

[23] Trzeciak S (2007). Early microcirculatory perfusion derangements in patients with severe sepsis and septic shock: relationship to hemodynamics, oxygen transport, and survival. Ann. Emerg. Med..

[24] Parežnik R, Knezevic R, Voga G, Podbregar M (2006). Changes in muscle tissue oxygenation during stagnant ischemia in septic patients. Intensive Care Med..

[25] Doerschug KC, Delsing AS, Schmidt GA, Haynes WG (2007). Impairments in microvascular reactivity are related to organ failure in human sepsis. Am. J. Physiol. Circ. Physiol..

[26] Mendelson AA (2020). Dynamic tracking of microvascular hemoglobin content for continuous perfusion monitoring in the intensive care unit: pilot feasibility study. J. Clin. Monit. Comput..

[27] Kilkenny C, Browne WJ, Cuthill IC, Emerson M, Altman DG (2010). Improving bioscience research reporting: the ARRIVE guidelines for reporting animal research. PLoS Biol..

[28] Tyml K, Budreau CH (1991). A new preparation of rat extensor digitorum longus muscle for intravital investigation of the microcirculation. Int. J. Microcirc..

[29] Akerstrom T (2020). Hyperinsulinemia does not cause de novo capillary recruitment in rat skeletal muscle. Microcirculation.

[30] Whitehead SN, Hachinski VC, Cechetto DF (2005). Interaction between a rat model of cerebral ischemia and β-amyloid toxicity. Stroke.

[31] Japee SA, Ellis CG, Pittman RN (2004). Flow visualization tools for image analysis of capillary networks. Microcirculation.

[32] Fraser GM, Milkovich S, Goldman D, Ellis CG (2012). Mapping 3-D functional capillary geometry in rat skeletal muscle in vivo. Am. J. Physiol. Circ. Physiol..

[33] Fraser GM, Goldman D, Ellis CG (2012). Microvascular flow modeling using in vivo hemodynamic measurements in reconstructed 3D capillary networks. Microcirculation.

[34] Zhai X (2018). Lactate as a potential biomarker of sepsis in a rat cecal ligation and puncture model. Mediators Inflamm..

[35] Ondiveeran HK, Fox-Robichaud AE (2004). Pentastarch in a balanced solution reduces hepatic leukocyte recruitment in early sepsis. Microcirculation.

[36] Polat G, Ugan RA, Cadirci E, Halici Z (2017). Sepsis and septic shock: current treatment strategies and new approaches. Eurasian J. Med..

[37] Tyml K, Yu J, McCormack DG (1998). Capillary and arteriolar responses to local vasodilators are impaired in a rat model of sepsis. J. Appl. Physiol..

[38] Singer M (2014). The role of mitochondrial dysfunction in sepsis-induced multi-organ failure. Virulence.

[39] Piper RD (1998). Leukocyte activation and flow behavior in rat skeletal muscle in sepsis. Am. J. Respir. Crit. Care Med..

[40] Madoiwa S (2015). Recent advances in disseminated intravascular coagulation: endothelial cells and fibrinolysis in sepsis-induced DIC. J. Intensive Care.

[41] Secor D (2010). Impaired microvascular perfusion in sepsis requires activated coagulation and P-selectin-mediated platelet adhesion in capillaries. Intensive Care Med..

[42] Tyml K (2011). Role of connexins in microvascular dysfunction during inflammation. Can. J. Physiol. Pharmacol..

[43] Tyml K, Wang X, Lidington D, Ouellette Y (2001). Lipopolysaccharide reduces intercellular coupling in vitro and arteriolar conducted response in vivo. Am. J. Physiol. Circ. Physiol..

[44] Lidington D, Ouellette Y, Li F, Tyml K (2003). Conducted vasoconstriction is reduced in a mouse model of sepsis. J. Vasc. Res..

[45] Mckinnon R (2006). Reduced arteriolar conducted vasoconstriction in septic mouse cremaster muscle is mediated by nNOS-derived NO. Cardiovasc. Res..

[46] Jagger JE, Bateman RM, Ellsworth ML, Ellis CG (2001). Role of erythrocyte in regulating local O_2_ delivery mediated by hemoglobin oxygenation. Am. J. Physiol. Circ. Physiol..

[47] You J, Johnson TD, Marrelli SP, Bryan RM (1999). Functional heterogeneity of endothelial P2 purinoceptors in the cerebrovascular tree of the rat. Am. J. Physiol. Circ. Physiol..

[48] Sprague RS, Ellsworth ML, Stephenson AH, Kleinhenz ME, Lonigro AJ (1998). Deformation-induced ATP release from red blood cells requires CFTR activity. Am. J. Physiol. Circ. Physiol..

[49] Wan J, Ristenpart WD, Stone HA (2008). Dynamics of shear-induced ATP release from red blood cells. Proc. Natl. Acad. Sci..

[50] Bateman RM (2001). Erythrocyte deformability is a nitric oxide-mediated factor in decreased capillary density during sepsis. Am. J. Physiol. Circ. Physiol..

[51] Emerson GG, Segal SS (1997). Alignment of microvascular units along skeletal muscle fibers of hamster retractor. J. Appl. Physiol..

[52] Murrant CL, Fletcher NM, Fitzpatrick EJH, Gee KS (2021). Do skeletal muscle motor units and microvascular units align to help match blood flow to metabolic demand?. Eur. J. Appl. Physiol..

[53] Mendelson AA (2021). The capillary fascicle in skeletal muscle: structural and functional physiology of RBC distribution in capillary networks. J. Physiol..

[54] Fujishima S (2016). Infection site is predictive of outcome in acute lung injury associated with severe sepsis and septic shock. Respirology.

[55] Shrum B (2014). A robust scoring system to evaluate sepsis severity in an animal model. BMC Res. Notes.

[56] Feng Q (2019). Characterization of sepsis and sepsis-associated encephalopathy. J. Intensive Care Med..

[57] Iwashyna TJ, Ely EW, Smith DM, Langa KM (2010). Long-term cognitive impairment and functional disability among survivors of severe sepsis. JAMA.

[58] Andonegui G (2018). Targeting inflammatory monocytes in sepsis-associated encephalopathy and long-term cognitive impairment. JCI Insight.

[59] Westhoff D (2019). Systemic infection and microglia activation: a prospective postmortem study in sepsis patients. Immun. Ageing.

[60] Hernandes MS (2014). The role of Nox2-derived ROS in the development of cognitive impairment after sepsis. J. Neuroinflammation.

[61] Ehlenbach WJ, Sonnen JA, Montine TJ, Larson EB (2019). Association between sepsis and microvascular brain injury. Crit. Care Med..

[62] Slessarev M, Mahmoud O, McIntyre CW, Ellis CG (2021). Cerebral blood flow deviations in critically ill patients: potential insult contributing to ischemic and hyperemic injury. Front. Med..

[63] Zong M-M (2019). Activation of β_2_-adrenoceptor attenuates sepsis-induced hippocampus-dependent cognitive impairments by reversing neuroinflammation and synaptic abnormalities. Front. Cell. Neurosci..

[64] Hoogland ICM, Houbolt C, van Westerloo DJ, van Gool WA, van de Beek D (2015). Systemic inflammation and microglial activation: systematic review of animal experiments. J. Neuroinflammation.

[65] Henry CJ, Huang Y, Wynne AM, Godbout JP (2009). Peripheral lipopolysaccharide (LPS) challenge promotes microglial hyperactivity in aged mice that is associated with exaggerated induction of both pro-inflammatory IL-1β and anti-inflammatory IL-10 cytokines. Brain. Behav. Immun..

[66] Qin L (2007). Systemic LPS causes chronic neuroinflammation and progressive neurodegeneration. Glia.

[67] Ha SK (2012). Acacetin attenuates neuroinflammation via regulation the response to LPS stimuli in vitro and in vivo. Neurochem. Res..

[68] Nishioku T (2009). Detachment of brain pericytes from the basal lamina is involved in disruption of the blood-brain barrier caused by lipopolysaccharide-induced sepsis in mice. Cell. Mol. Neurobiol..

[69] Masocha W (2009). Systemic lipopolysaccharide (LPS)-induced microglial activation results in different temporal reduction of CD200 and CD200 receptor gene expression in the brain. J. Neuroimmunol..

[70] Hoogland ICM (2018). Microglial activation after systemic stimulation with lipopolysaccharide and *Escherichia coli*. Front. Cell. Neurosci..

[71] Semmler A, Okulla T, Sastre M, Dumitrescu-Ozimek L, Heneka MT (2005). Systemic inflammation induces apoptosis with variable vulnerability of different brain regions. J. Chem. Neuroanat..

[72] De Blasi RA (2005). Microvascular dysfunction and skeletal muscle oxygenation assessed by phase-modulation near-infrared spectroscopy in patients with septic shock. Intensive Care Med..

