# Supplementary material for: Spectroscopy detects skeletal muscle microvascular dysfunction during onset of sepsis in a rat fecal peritonitis model
Source: Sci Rep. 2022 Apr 15;12:6339. doi: 10.1038/s41598-022-10208-w (PMC9012880; doi:10.1038/s41598-022-10208-w)
Supplement: Supplementary file 1 — Supplementary Information. [file 41598_2022_10208_MOESM1_ESM.pdf]

Spectroscopy detects skeletal muscle microvascular dysfunction during onset of sepsis in a rat fecal peritonitis model

Paulina M. Kowalewska[1], Justin E. Kowalewski[2], Stephanie L. Milkovich[1], Richard J. Sové[3], Lin Wang[4], Shawn N. Whitehead[4] and Christopher G. Ellis[1*]

[1]Department of Medical Biophysics, University of Western Ontario, London, ON, Canada

[2]Department of Psychiatry, University of Western Ontario, London, ON, Canada

[3]Department of Biomedical Engineering, Johns Hopkins School of Medicine, Baltimore, MD, USA

[4]Department of Anatomy and Cell Biology, University of Western Ontario, London, ON, Canada

SUPPLEMENTARY FIGURE

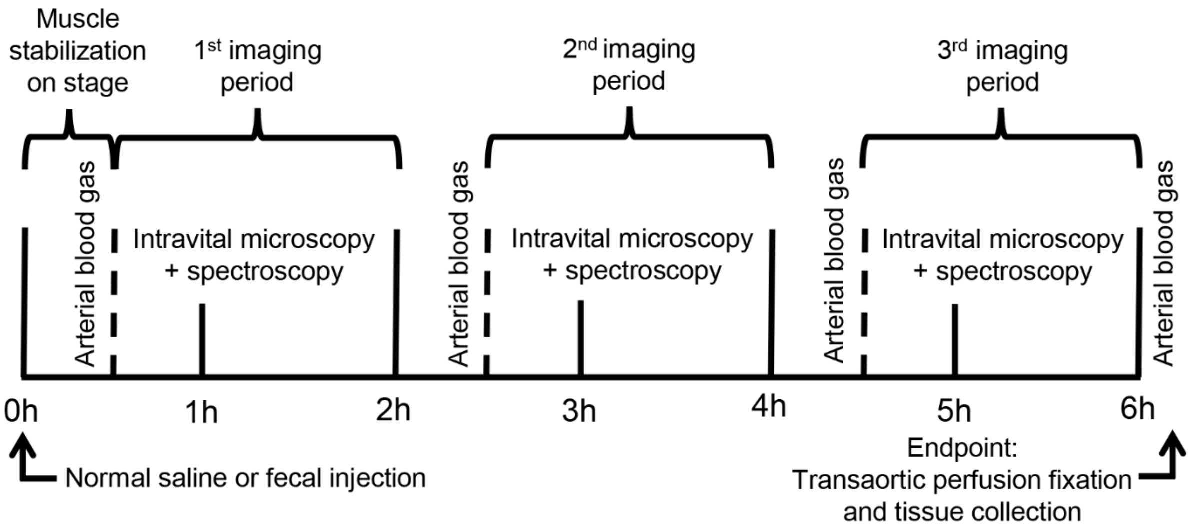

Supplementary Figure S1. Experimental design. After intraperitoneal injection of normal saline (control) or a fecal slurry (feces-induced peritonitis, FIP), the extensor digitorum longus muscle was stabilized on the microscope stage for 30 min. Intravital video microscopy (IVVM) and continuous wave spectroscopy recordings were taken over three 1.5-hour blocks of time. Arterial blood gas measurements were taken periodically. Mean arterial pressure was measured over the entire 6-hour period. At the endpoint, animals underwent perfusion-fixation and organs were collected. IVVM and spectroscopy were done with 5 FIP and 5 control rats. Additional IVVM recordings with organ collection were done in a separate group of control and FIP rats (n = 5).
